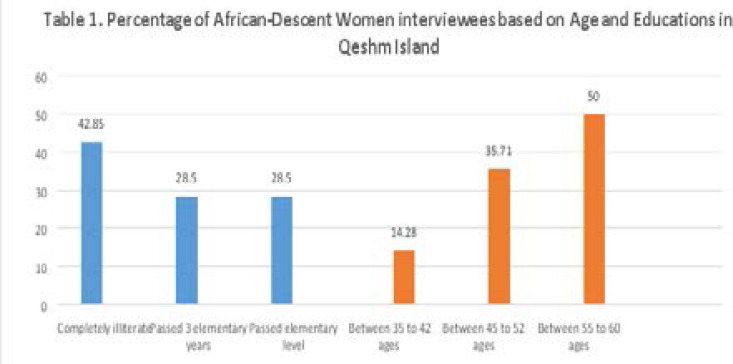# A Study of Female Genital Mutilation of African-Descent Iranians in Qeshm Island

**DOI:** 10.4314/ahs.v22i1.9

**Published:** 2022-03

**Authors:** Arabahmadi Amirbahram

**Affiliations:** University of Tehran

**Keywords:** Female Genital Mutilation, Gender Discrimination, Human Rights violation, NGOs' Campaign, Qeshm Island

## Abstract

**Background:**

This article investigates the practice of female genital mutilation (FGM) as a long-held custom in Qeshm Island, which makes many African-descended women face different physical and psychological health problems.

**Objective:**

To investigate the prevalence of female genital mutilation in Qeshm Island and the traditional mode of thinking of Afro-Iranian people of the Island about this practice.

**Methods:**

This study is based on the descriptive analysis method. The questions of the study are (a) Why female genital mutilation is still practiced in Qeshm Island; (b) What are the mental and physical effects of female genital mutilation on women; and (c) How government or NGOs are fighting against this tradition.

**The results:**

This article has found out that female genital mutilation resulted in many lifelong diseases and sexual degradation in African-descended women of Qeshm Island. This article also illustrates that the best way to combat this wrong tradition is to inform people by gradual training without any insult to their beliefs.

**Conclusion:**

This study reveals the prevalence of a false tradition and the necessity of behavioral change. In doing so, the government and NGOs' strong actions and attracting the support of the community elders are also needed.

## Introduction and Aim

Female genital mutilation, sometimes called “Female Cutting” or “Female Genital Cutting” 1 is an ancient custom that originated from North Africa and Egypt, particularly during Ancient Egypt 2. This tradition is still widespread in many ethnic groups in some parts of the world and mostly in Africa. 3 According to the World Health Organization, this operation has been documented in many countries, mainly in thirty African countries 4, Middle East, Asia countries, and among some migrant communities in North America, Australia, and Europe 5. Approximately 200 million women worldwide have experienced this procedure. There are an estimated 3 million girls at risk of undergoing female genital mutilation every year 6.

Generally speaking, FGM is cutting part of the female genital, usually without any sanity 7, to observe old ethnic customs of tribes and usually in traditional styles.^8^ Female genital mutilation is typically done by cutting a part of the girl's genital 9,10.

FGM carry out on young girls between ages 1 to 9 in countries where it is practiced 11 rarely in adulthood. Although it cannot be precisely said infant and underage girls are victims of FGM, any girl from her birth to maturity is in danger of being circumcised. 12 Some of female genital circumcision is operated by trained nurses and sanitary equipment, yet many others are done by traditional older women and tribal midwives. 10 13 A literature review of this subject illustrates that except for one book entitled: “Razor & Tradition,” so far, no scholarly articles have been written on female genital mutilation among Afro-Iranians in Qeshm Island. Therefore, this paper is novel in examining FGM as an old tradition. This paper also explains the difference of opinions between new and old generations of the African-descent community of Qeshm Island for either preserving or abandoning FGM.

Iran is one of the Asian countries where FGM is practiced. 14 Although there is no precise statics about the percentage of circumcised girls, the FGM prevalence in Iran is low compared to other countries in the Middle East and Africa. Based on available sources, FGM is mainly carried out in four provinces, including Kurdistan, Kermanshah, West Azerbaijan, and Hormozgan^15^,^16^,^17^, while it is more prevalent in Qeshm Island in the southern province of Hormozgan 18 than in any other parts of Iran. Qeshm is the largest Island in Iran, including several cities and dozens of villages, located in the Persian Gulf and the Strait of Hormuz. Due to its location in the important region of the Persian Gulf and the Strait of Hormuz, Qeshm has a special strategic position. With 1491 square kilometers, population distribution on Qeshm Island is 153000 in 2020 19. Most of the island's industries are related to marine, ecotourism, oil and gas, energy and environmental industries. The island also has unique features in terms of indigenous culture such as traditional architecture, historical monuments, celebrations and folk ceremonies as well as indigenous industries. Qeshm Free Zone Organization Supervises and implements all industrial projects and socio-cultural activities. 20

This Island has significant ethnic diversity, and in addition to indigenous Iranians, some of the African descent people live there. The arrival of Africans from East and Southeast Africa to Iran dates back to three centuries ago, mainly during the eighteenth and nineteenth centuries. Africans were brought as slaves. 21, pp. 55–6 22 After the abolishment of Slavery, African slaves in Iran gradually obtained their freedom. Many of them live in Tehran and other cities, and they are completely assimilated into the host population and have disappeared as a different ethnic group. Southern Iran is the only part of the country in which Afro-Iranians live as ethnic communities. The total numbers of imported African slaves differed among the regions. Perhaps this difference was one of the significant reasons for such complete social assimilation. Thus, most of the slaves were retained in the south of Iran, and they were involved in various economic sectors and created the Afro-Iranian communities in the region. 22 Qeshm Island was one of the areas where Africans gradually formed a community, and they have been living in some villages on the Island ever since. Though there is no accurate statics of their population, approximately 5000 African-descents live in rural areas around Qeshm County. 23 Afro-Iranian communities preserved their ancestral customs, therefore despite cultural and social interactions and assimilation with Iranians, they preserved some parts of their traditions. Female Genital Mutilation known as” Muslim razor” or “Sunnh” 24 is one of the old customs that Iranians of African-descent practice in the southern coasts and islands of Iran -including Qeshm Island -. According to unconfirmed statistics, 90 to 95% of African girls in Qeshm Island are circumcised by local midwives between ages 1 to 9, which is the highest rate of its kind among local communities in the region. 23

Since FGM is an integral part of the African descent community's traditions in Qeshm Island - who have preserved the tradition- this small community is chosen as the case study for this research. This paper takes to investigate this tradition among the African-descent community in Qeshm and its current practice implications. The main hypothesis says that persistence on implementation of old traditions among Afro-Iranians in Qeshm Island is the main reason for the continuation of FGM in the Island. The main research questions addressed here are (a) Why Female Genital Mutilation is still practiced among the African-descent community in Qeshm Island? (b) What are the mental and physical consequences of female genital mutilation for Afro-Iranian women? And (c) How Government and non-government entities, are combating this tradition?

## Data and Methods

Data: The data presented in this article was extracted from the content analysis of available documents as well as investigation, validation, and codification of questionnaires.The data are divided into three parts:

1. Most of the data is based on the analysis of the responses to the questionnaires. The sample was chosen from Afro-Iranian women in three selected villages of Qeshm Island
2. Some of the information was gathered through interviewing several medical female doctors and midwives in three rural medical centers about the effects of Female Genital Mutilation on Afro-Iranian women of the Island
3. The researcher also used some books, scholarly papers, and credible databases.

There was a total of 80 participants in this study including seventy African-descents and ten medical staff. Afro-descents women were from the villages of “Suza,” “Table,” and “Dargahan” With very little education. 30 of them were completely illiterate whilst 20 had passed 3 elementary years only. The rest of 20 had passed elementary level. Participants' ages varied between 35 and 60 years old. 10 of them were between 35 to 42 ages, 25 of them were from 45 to 52 years old and the rest of 35 were from 55 to 60 years old and due to this age differences, had different views on FGM and its complications on those who have undergone the Sunnah. All of them were married, had children and had experienced FGM. Participants were assured that we would endeavor to keep their identities hidden and were requested to give accurate information to the best of their knowledge. Collected information indicates that there are misconceptions about the “clitoris” among the Afrian-descent women as a male organ, and negative responces of respondents, show their utterly hidden beliefs against uncut women as not appropriate mothers and wives. Most of these negative opinions are based on narratives of elders that are not necessarily experiential. Additionally, most African-descent people of Qeshm Island believe that circumcision starts girls' puberty process, and it makes them ready for marriage, and having a family. Furthermore, African-descent elders in Qeshm believe that most Afro-Iranian young men, based on their traditional principles, are unwilling to marry uncircumcised girls and consider them impure. 25

Another part of the information was obtained from interviews with 10 women staff of medical clinics located in the villages of “Suza,” “Tabl,” and “Dargahan.”

These people's education levels ranged from bachelor to doctorate degrees, and their ages varied between 35 and 45 years. Six of them were married, and four were single. All of them gained experiences because of the continuous interactions with African women, and had a good knowledge of FGM, and provided significant information about the physical and mental effects of circumcision. The information they offered of psychological effects was precious.

## Methods

This study is a cross-sectional descriptive content analysis study carried out November 12th to December 14^th^, 2020 on 70 local Afro-Iranian females as well as 10 originally-Iranian female nurses or doctors, in some villages in the Qeshm Island. Medical centers were selected as the sampling frame of the study. Multistage sampling was used, and three medical centers in Qeshm Island were randomly selected. Then, convenience sampling was used to select the required number of participants from each stratum. The paper used the method of descriptive content analysis. The descriptive content analysis identifies and designates the main content of the data, chronologically, thematically, or otherwise, either subject-focused or researcher-focused, and encompasses counting, listing, practicalizing, classifying assessment, and clarification. 26 Based on the above-mentioned method, all the information was collected through questionnaires, consisting of questions for gathering information from some random respondents. Some valuable evidence has been collected through interviews with a few selected female health cadres, whilst the research does reveal any participant's identity and does not mention them anonymously. Although it seems that the methodology used in this study was quite appropriate; but the severe lack of information, especially written sources, as well as the strong refusal and reluctance of the interviewees to talk and provide any information about FGM and its physical symptoms and considering it as a violation of their privacy was one of the challenges that was eventually finalized.

## Findings and Discussion

There is no doubt that FGM accompanies by an extensive variety of temporary and long-held physical, psychological, sexual, and obstetric health risks 27.

Types of Female Genital Mutilation in Qeshm Island: Although based on the World Health organization, generally, there are four types of Female Genital Mutilation in the world 1 including: I Clitoridectomy, II excision, III infibulation and IV – all other harmful procedures^28^ but the most common type of Female Genital Mutilation in Iran including Qeshm Island 29 is mainly type number one -Clitoridectomy-(Partial or total removal of the clitoris and/or the prepuce or the mildest type) 30. According to the questionnaires' extracted data, more than half of the circumcised girls and females expressed that only part of their clitoris has been amputated. The rest of them have mentioned that only the clitoral hood has been removed. Of course, during the last decade, as a result of the enlightened actions of some NGOs, female genital mutilation among the natives - including African descents of Qeshm Island - has been reduced to merely using the razor blade on the clitoris and drawing a drop of blood called “Muslim blood” 31 or just cut with the razor. 18 Those who perform this circumcision, known as “Khatans,” believe that bleeding makes girls clean and pure. 32

### Physical complications

Based on Unicef 2021 report, child marriage specially those who have already been circumcised threaten the well-being of millions of girls around the world 33 and can cause severe bleeding, problems urinating, and later cysts, infections, as well as complications in childbirth and increased risk of new-born deaths. 34 Circumcised girls in Qeshm Island experience mental and physical health problems – but not as severe as African countries - like infection, uterus cyst, painful sexual intercourse, childbirth difficulties, repetitious urine, consistent bleedings, and some other physical and mental problems 32. Other physical problems mentioned by respondents include severe period pain, bleed, and infection during pregnancy. These symptoms are common for Afro-Iranian women in Qeshm Island who have experienced the first type of circumcision (Clitoridectomy).

### Psychological Complications

Circumcised Afro-Iranian women in Qeshm Island experience physical problems and face mental health problems. Some of the health providers in Qeshm Island's clinics mentioned that anxiety, permanent phobia, low self-confidence, and low self-esteem are some of the mental health problems of circumcised African-descended women on Qeshm Island. Furthermore, some Afro-Iranian families in Qeshm Island – who are suffering from poverty and lack of proper education - believe that their girls must get married immediately after puberty to avoid any sexual perversion. Among these families, many girls are married between the ages of 12 and 14 while they are not physically or psychologically ready, which usually causes some mental disorders for them in the long run.

### Provided Justifications for the Practice

Mostly based on extracted information as well as available sources; African ancestral cultures, family associations, social circumstances, and lack of education are some provided justifications for circumcision among Afro-Iranian families in Qeshm Island. 32 One of the important motives of African-descent families for circumcising their daughters is that they think she can't have a good husband if she is not circumcised. 35 Furthermore, there is a common belief that by the circumcision of young girls, they could properly control their sexual desires after maturity. 36 Furthermore, their parents believe that there is dirty blood insides the uncircumcised girls' body, so by circumcision the girls get rid of dirty blood, and their body becomes clean. 37

Most Afro-Iranian girls are raised to believe that mutilation is not harmful but is in their favor and for having a better future. 23 Based on what the circumcised girls learn from their grandmother and mothers, they convincingly accept circumcision, even if they had the power to reject this tradition's implementation; since they believe that they may encounter social and physical challenges in their adulthood, mostly after marriage. 14 The other point which is popular among local leaders and elders of Qeshm' African-descent families is the concept of purity. They do believe uncircumcised girls are not clean. Therefore, any interaction with them, even getting a glass of water or food, is not advisable, and only after circumcision, the girls will receive traditional legitimacy and would be considered as “Halal” and pure. 38

## Results

The data presented in this article was extracted from the content analysis of available documents as well as investigation, validation, and codification of questionnaires. The data are divided into three parts:

1. Most of the data is based on the analysis of the responses to the questionnaires. The sample was chosen from Afro-Iranian women in three selected villages of Qeshm Island
2. Some of the information was gathered through interviewing several medical female doctors and midwives in three rural medical centers about the effects of Female Genital Mutilation on Afro-Iranian women of the Island
3. The researcher also used some books, scholarly papers, and credible databases.

### Questionnaires

As mentioned, some of the data were collected by using a five-part researcher-made questionnaire. The first table includes questions about demographic information, including age and educations.

The second part of the questionnaire asks about their knowledge of FGM's benefits, and the third part evaluates Afro-Iranian women's general opinion about the continuation of FGM. Responses to these questions were combined and presented in Tables 2 and 3. All of the participants had no work experience. More than 75% of participants believe that FGM controls sexual desires after puberty. 60% of participants think FGM is necessary to preserve tradition. A majority of the participants (85%) believe that FGM is crucial for having a good marriage; 90% of the participants emphasized that FGM preserve virginity. Half of the participants (50%) believe that FGM cleans the body of dirty blood, and circumcised women don't have any dirty blood in their bodies. 75% of participants believe that FGM provides social dignity for women.

**Figure:**
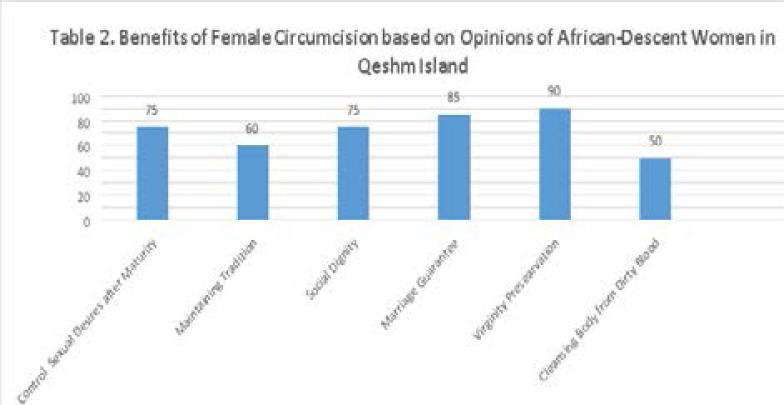


**Figure:**
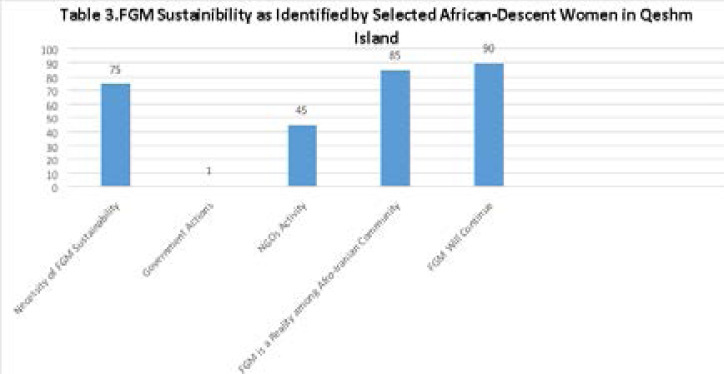


Table 3 presents the results of the African-descent women's responses to the third part of the questionnaire about the continuity of FGM. A relative majority of the participants (75%) expressed that FGM should continue. Almost none of the respondents had any idea that the government has the policy to fight FGM. Some participants confirmed the activity of NGOs (45%), and 85% believed that FGM is a reality among the Afro-Iranian community on the Island. 90% of respondents insisted on the preservation of FGM as a portion of their old traditions.

Table 4 presents the responses to the fourth part of the questionnaire, which is about the physical complications of FGM. 15% of participants acknowledged that they have suffered from infection or uterus cyst, while 90% of them exposed that they had or still have pain during intercourse. An absolute majority of the participants (96%) revealed they had experienced severe pain in childbirth and 23% of them stated they have little repetitious urine. Consistent bleedings were confirmed by 9% of participants, and 13% of selected respondents verified other physical problems caused by circumcision.

**Figure:**
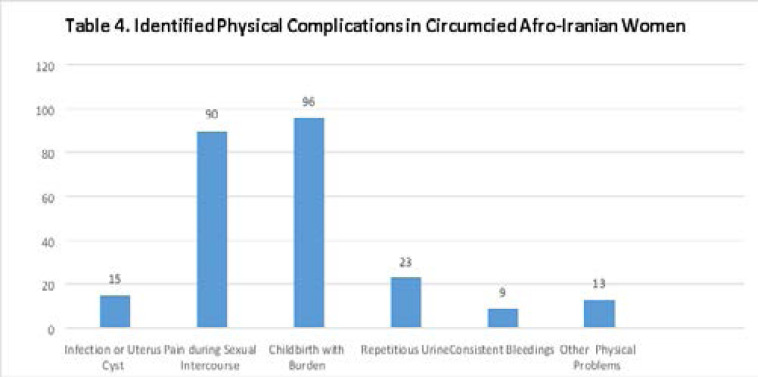


The fifth part of the data extracted from interviews with a number of educated women in Qeshm Island who work as doctors and nurses in Qeshm's medical centers. The questions and answers were mainly related to the mental health of circumcised women. Based on the mentioned interviews, lower self-esteem and lower self-confidence than the uncircumcised girls, feeling nervous, constant phobia, and anxiety disorder are permanent mental complications that circumcised Afro-Iranian girls experienced throughout all their lives. Table 5 demonstrates the identified psychological complications of circumcised Afro-Iranian girls based on interviews with female medical staff.

**Figure:**
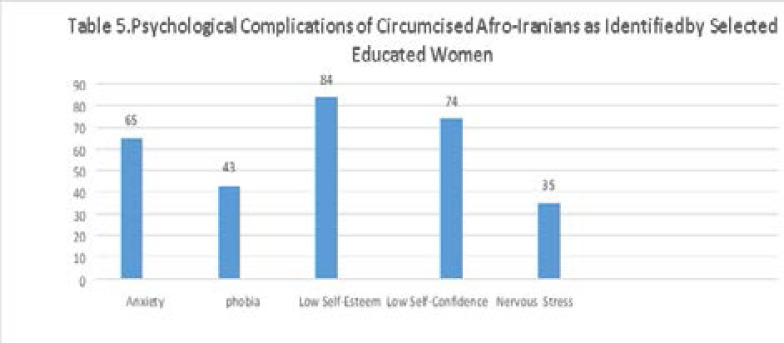


### Anti FGM Campaign

#### Government

Female Genital Mutilation has not yet been outlawed in the Islamic Republic of Iran, and talking about it in public is considered taboo.^39^ The central government and local offices are not active in this field, and Iranian Health organizations also do not deal with FGM and its dimensions. 40 Although, over the past years, the Department for Women and Family Affairs of President has been trying to pass laws to ban Female Genital Mutilation through the Islamic Consultative Assembly, and some unofficial steps were taken by the Presidential Adviser on Women's Affairs as well as National Association of Children's Rights in the Ministry of Justice; 35 however in practice, there are no laws that penalize those who circumcise women, including Qeshm Island. Of course, some laws of the Islamic Penal Code of the Islamic Republic of Iran impose indirect penalties for those who practice Female Genital Mutilation:

- Article 386 criminalizes the mutilation of the body. If the crime were intentional, it would be punished according to the retaliation (Qisas) law – the perpetrator shall be given a sentence that is equal to the crime committed. If the crime was unintentional, the penalty might be either Diya (monetary compensation or blood money) as prescribed by Islamic law for that particular crime, or “Ta'zir” (where Islamic law does not prescribe a punishment for a particular crime, the punishment is left to the discretion of the judge). 41
- Article 479: If a woman's genital is totally severed, she shall be entitled to her full blood money, and if only half of her genital is severed, half of her blood money is due her. 42
- Article 664: Cutting or removing each of the two sides of the female genitalia leads to “Diya” (compensation) equal to half of the full amount of “Diya” for a woman's life.
- Article 706: Elimination of the power of ejaculation or male reproduction or female pregnancy or eliminate the pleasure of the sexual ability of a man or woman causes compensation (Diya or blood money)
- Article 707: Complete elimination of the power of intercourse leads to complete blood money. 41

As already mentioned, there is no direct law against FGM in Iran. On the other hand, Iran has accepted the convention on the rights of persons with disabilities, and FGM has been considered a disability, and its practice should be stopped. 43

#### NGOs

Since Female Genital Mutilation is still taboo in Iran -including in Qeshm Island- and the public doesn't have enough information about it, the NGOs' activities in this area are slow, gradual, and limited. One of the important non-governmental organizations that has been active in this issue during the last decade is the “Step by Step to Stop Female Genital Mutilation Campaign,” and Qeshm Island has been one of the focus areas of the campaign. Activities of this campaign include talking to heads of local communities (Sheikhs) in Qeshm, meeting elderly women who have become so-called Sunnah (been circumcised), meeting traditional female circumcisers known as “Khatan,” the publication of several books and articles, and set up some educational workshops. As a result of this campaign's activities, the percentage of Female Genital Mutilations on the Island has decreased compared to the past ten years, especially since the campaign leaders gained the trust of indigenous people, including Afro-Iranian women.

### Challenges and Difficulties

One of the problems in combating Female Genital Mutilation in Qeshm Island is the secrecy of the FGM procedure, which is usually performed at home and secretly by local midwives. In addition, the tradition of female genital mutilation is still known as taboo among Qeshm' African-descent and is less discussed in societies. The concealment of this FGM procedure also means that there are no reliable statistics about it. On the other hand, there are no reliable resources for comprehensive pathological research for responsible institutions, especially for the Ministry of Health and Medical Education and the Welfare Organization on Qeshm Island. Local officials usually avoid addressing this issue and even avoid using the word Female Genital Mutilation, which they consider a red line.

Since there is no prohibition or restriction on Female Genital Mutilation in Iran, there are no specific penalties for those who circumcise their wives and daughters. Medical staff in Qeshm Island have not yet been specifically trained in the dangers of FGM. Despite some measures by NGOs, government officials have not yet acted to raise public awareness about Female Genital Mutilation. Also, many African-descent families in Qeshm Island still consider uncircumcised girls unclean. They believe that only if they circumcise them they will be clean and they will have a promising future and good marriage.

Other obstacles and challenges include – but are not limited to - illiteracy and lack of education among traditional families, social prejudices, and commitment to customs and traditions among African-descent people of Qeshm Island.

## Conclusion

Fortunately, during the last decade, significant actions in controlling and reducing female genital circumcision have been taken in different parts of Iran -in particular Qeshm Island- which hold promise for a better future on stopping this sociocultural custom. Field studies, as well as available information and published data by some NGOs, indicate that due to the efforts of NGOs and the support of some religious authorities, the number of FGM operations in Iran is continuing to decrease. 29

As a result, it is possible that in the next years, Female Genital Mutilation will be announced outlaw by the Iranian parliament. Yet, to eliminate this social contract from Iran – in provinces of Kurdistan, Kermanshah, West Azerbaijan, and Hormozgan, and among the African-descent community in Qeshm Island, which is the case study of this paper- a strong platform is needed for change in the behaviors, viewpoints, and values of ethnic groups. Some researches carried out about FGM in Iran suggest that the best remedy for the abolishment of Female Genital Mutilation is to convince the central government to declare it an illegal act as well as the establishment of close cooperation with the traditional communities, elders, and local leaders where FGM is still prevalent.

While concentrating on the African-descent community in Qeshm Island, social activists and NGOs who are campaigning against Female Genital Mutilation in the Island should first take time to know this ethnic group and should avoid policies that may be perceived by the people in that region as aggressive toward their traditions and cultural protocols. Due to African-descent tradition, they need a longer time to accept the realities of this harmful tradition.

An important consideration of FGM practice in Qeshm Island is the parent's persistence to mutilate their daughters; they believe it will protect and provide a promising future for their girls. In fact, family dignity and social situation play an influential role in preserving FGM among African-descent families, which makes it difficult for them to end the practice. Even after parents realize that FGM can cause severe damage, they will keep it up because they are afraid of their daughters' future, repercussions, and ethical judgments of society's expectations.

For the African-descent community of Qeshm, failure to imitate FGM leads to social segregation, exclusion, displeasure, reproach, or even ferocity – as well as having an outcome on a girl's marriageability. Therefore FGM is still considered a social norm that members have to follow it. Although Afro-Iranians of Qeshm are somehow aware of FGM's dangerous effects, Female Genital Mutilation is a part of their ancestral culture. Any strategy to combat it should be done gradually and with caution.

Indeed, the most important task to fight against FGM among Afro-Iranians of the Qeshm Island is understanding the traditions of these people, at the same time broadcasting the detrimental consequences of some customs that have to be either updated or set aside, while FGM is on the top of them. Therefore, any action against FGM as entering a sensitive and private arena must be taken cautiously based on a thorough knowledge of African-descent customs and knowing their women's pre-existing assumptions. Also, their gradual enlightenment should be done via the existing framework of tribal chiefs. Fighting FGM needs patience, wisdom, and an understanding of Afro-Iranian traditions directly connected to female destiny. Interestingly, apart from African-descent fathers who insist on circumcision of their girls, mothers who have already experienced this awful operation will follow their husbands and do expressed that they intend to subject their daughters to FGM to guarantee their futures and avoid the likely dishonor and disgrace that would encompass them if it doesn't happen.

Therefore, the author suggests that the fight against Female Genital Mutilation -at least among the Afro-Iranians community of Qeshm Island -requires enduring, long-term planning, and local administrators' mobilization NGOs, health centers, religious leaders, freelance volunteers, social activists, and women's associations. It is also important to emphasize that any strategy toward combatting FGM regarding African-descent people should not be perceived by people as aggressive toward their traditional and cultural protocols, while it has to be done through gradual culture building and understanding.

In addition, nurses working in urban and rural hospitals and clinics must be trained to inform African-descent families in Qeshm. They have to try to play an incisive role to reduce the percentage of circumcisions in this traditional society.

Fortunately, over the past decade, in addition to some grand Iranian Shiite Ulama who have stated their opposition to Female Genital Mutilation, Iranian Sunni scholars have also opposed Female Genital Mutilation by issuing statements that have had a positive effect on the changing views of local communities whose still insist on continuing the tradition.

Of course, over the past decade, with the increase in the number of the educated female of African-descent in Qeshm Island, an increase in the use of social networks such as “Instagram” and “WhatsApp,” and an increase in reading articles about the consequences of female genital mutilation, a gap has emerged among women across the generations. The older un-educated generation continues to emphasize the preservation of circumcision as an old tradition and believes it would guarantee the families' survival; while young educated women consider circumcision a violation of women's rights. They consider FGM a tradition, harmful to the physical and mental health of girls, and they believe that this tradition should be abolished. This duality indicates that not in a distant future, with the death of the elders of this society, FGM will gradually be abandoned in this community. So, through enlightening and increasing the number of educated women in the community, definitely in the future, there would be a fundamental behavioral change among the next generation of Afro-Iranians of Qeshm to either reduce or abandon it.

## Recommendations

1. Enforcement of national laws prohibiting FGM in the Islamic Consultative Assembly of Iran;
2. Implementation of Islamic penal code for female mutilation;
3. Add contents about FGM in medical and paramedical courses to let graduates learn more about this tradition;
4. Culture building by raising awareness of traditional communities' leaders to abandon some of the harmful old traditions, including FGM;
5. Teaching and warning midwives about the dangers of circumcising for girls with the help of local health officials;
6. Production of educational programs to broadcast on radio and television for explaining the long-term physical and mental dangers of FGM on women;
7. Collaboration of central government with UNESCO and UNICEF, to use their experience in combating FGM;
8. Engagement of NGOs against FGM through organizing educational seminars, workshops, and publishing books and pamphlets, in order to inform traditional communities about the urgent necessity of stopping FGM;
9. Improving the public knowledge about FGM and its health consequences.
10. Encouragement of the girls and women subjected to FGM, to seek healthcare;
11. Provision of healthcare, in the form of surgery or psychosexual consulting to the victims;
12. To establish specialist medical centers with HCPs who have experience of FGM, in the regions with high prevalence rate;

## Limitations

Remarkable information of the current study are extracted from questionnaires answered by several African-descent women of Qeshm Island who live in towns and villages. Therefore, outcomes neither present all Afro-Iranian families in Qeshm Island nor the experience of all African-descent circumcised women living on the Island. Female Genital Mutilation is still considered a cultural and social taboo among African-descent women of Qeshm especially when they want to express their feelings. They even consider FGM as an essential part of their ancestral culture that has to be preserved. Many of them were reluctant to answer the questionnaires provided to them by female nurses in several medical centers in Qeshm and the surrounding villages; they believed that we are violating their privacy by asking such questions. So, convincing them to participate was a big task and, to some extent, time-consuming. Clarifying the extracted results during the data analysis to have accurate results was another difficult task. Assuring them that their identity will be completely hidden was very problematic. Some of them afraid that if their identity reveals, they might face complications in their private lives. The scarcity of reliable sources and documents was another serious problem.

## Figures and Tables

**Figure:**